# Seismic microzonation of a region with complex surficial geology based on different site classification approaches

**DOI:** 10.1186/s40677-021-00198-8

**Published:** 2021-10-11

**Authors:** Mohammad Salsabili, Ali Saeidi, Alain Rouleau, Miroslav Nastev

**Affiliations:** 1 Département des Sciences Appliquées, Université du Québec à Chicoutimi, Saguenay, G7H 2B1 Canada; 2 Geological Survey of Canada, Quebec City, QC G1K 9A9 Canada

**Keywords:** Seismic microzonation, Building code, Shear-wave velocity, Fundamental site period

## Abstract

A seismic microzonation study was conducted to refine the seismic hazard model for the city of Saguenay, Canada. The Quaternary geology underlying Saguenay shows complex glacial and post-glacial stratigraphy with a number of buried valleys filled with fluvioglacial and glaciomarine sediments. High impedance contrast between rock formations and surficial sediments is prone to seismic amplification. To evaluate their applicability, advantages and limitations in capturing the geological specificity of the study area, four site classification methods were applied: the current National Building Code of Canada (NBCC) and Eurocode 8, both mainly based on the average shear-wave velocity for the surficial sediments (*V*_*S,avg*_) and for the top 30 m (*V*_*S,30*_); a method based on the fundamental site period (*T*_*0*_); and a hybrid method based on the combination of *V*_*S,30*_, *T*_*0*_ and *V*_*S,avg*_. The study specifically aimed to evaluate the importance of the site classification parameters on the resulting microzonation maps. *V*_*S,30*_ is capable to present the geological and geotechnical site conditions, however, the results may be further improved by considering *V*_*s,avg*_ in shallow and *T*_*0*_ in thick layers of soil sediments as secondary parameters. The *T*_*0*_ method gives also satisfactory results with *T*_*0*_ showing a better correlation to *V*_*s,30*_ than to *V*_*s,avg*_. The versatile hybrid method may be challenging to apply in certain cases with its nine different site categories and parameters.

## Introduction

An important aspect of geotechnical earthquake engineering is related to the evaluation of the expected intensity and the dominant period of the seismic ground motion at a given location. Knowledge of the geological and geotechnical properties of the surficial sediments is important in this respect since they tend to modify the amplitude and frequency content of the incoming seismic waves, a phenomenon known as the site effect (Seed et al. 1976). Significant damage to the built environment during the 1985 M8.0 Mexico City and 1989 M6.9 Loma Prieta earthquakes was attributed to the site effect (Borcherdt 1994). To improve its understanding, Borcherdt (1994) proposed a simplified empirical procedure for delineation of local site categories and associated amplification factors in terms of the time-averaged shear-wave velocity of the top 30 m, $V_{s,30}$. Ever since, $V_{s,30}$ has been adopted by building codes (e.g., BSSC 2015) to determine the seismic lateral forces generated by the ground motion.

The capacity of *V*_*S,30*_ as an efficient predictor of the local site amplification has also been criticized by several authors (e.g., Castellaro et al. 2008; Luzi et al. 2011; Braganza and Atkinson 2017; Pitilakis et al. 2018). It can particularly be questioned in regions such as Eastern Canada, which are characterised with significant impedance contrasts, where thick surficial sediments with relatively low *V*_*S,avg*_ overly stiff bedrock (Braganza et al. 2016; Braganza and Atkinson 2017). Under such conditions, the impedance contrast contributes to shorten the incoming shear-wave wavelengths and increasing their amplitudes (Hunter and Crow 2012). The amplification can further be increased when the shear waves become trapped in low-velocity valleys filled with fluvioglacial and glaciomarine sediments contributing to a resonance effect at the fundamental period (*T*_*0*_) until their energy is dissipated (Kramer 1996; Hunter and Crow 2012).

A standard site classification scheme considers site classes including hard rock, moderately fractured and weathered rock, stiff and dense unconsolidated soil, loose sandy soil, and soft clayey soil, each with its own range of $V_{s,30} ,V_{s,avg}$ and *T*_*0*_. Such classification provides a straightforward basis for mapping local site conditions in seismic microzonation studies (Bard and Riepl-Thomas 1999). The results of the seismic microzonation studies are usually presented on maps identifying and characterising zones with seismically homogeneous behaviour, e.g., zones susceptible to local seismic amplification or zones prone to instability, such as soil and rock sliding (Shano et al. 2020) and liquefaction (e.g., Huang et al. 2019). National and international guidelines propose a multi-step approach to conduct a seismic microzonation study (e.g., TC4-ISSMGE 1999; SM Working Group 2015). The seismic microzonation at grades I and II is dealing with qualitative and semi-quantitative evaluation of site classes and associated amplification. Here, surface and subsurface data are acquired from field tests and existing geological, geotechnical and geophysical maps as a basis to infer potential site amplification (Molnar et al. 2020). The grade III seismic microzonation complements results from grades I and II with detailed seismic site response analyses in terms of amplification of the ground motion using 1D and 2D numerical analyses (Licata et al. 2019). Within this framework, the seismic site classification based on building code provisions can be considered as a grade II seismic microzonation focusing on local amplification of seismic motion.

Some regions in the world, such as Eastern Canada, are characterised by significant impedance contrasts between the rock and soil deposits, and also show heterogeneous surficial geology units with variable thickness and stiffness properties (Braganza and Atkinson 2017). In this context, the objective of the present study is to refine the seismic hazard model for the city of Saguenay, a region in Eastern Canada characterised by irregular topography (i.e., valleys and hills), with highly heterogeneous surficial geology of variable thickness and stiffness properties as well as with high impedance contrasts between the rock and overlying soils. It is aiming to determine the relative importance of the main site effect parameters, i.e., *V*_s,30_, *V*_s,avg_, and *T*_*0*_, using 3D geological and geotechnical models. The specific objective is to determine the applicability of four site classification methods: the current NBCC (NRC 2015), Eurocode 8 (CEN 2004), the fundamental site period *T*_*0*_ (Zhao et al. 2006) and the approach developed by Pitilakis et al. (2018). First, a review of the four site classification schemes and the geological settings of the study area are presented. Then, seismic microzonation maps are generated applying each of the methods. The results of the comparative analysis are given at the end.

## Site classification schemes

Several standard site classification methods are largely used in seismiengineering. Normally, a standard site classification scheme considers hard rock, moderately fractured and weathered rock, stiff and dense soil, loose sandy soil, and soft clayey soil. To differentiate among the different site classes for the construction of new buildings and other structures, building codes such as the current NBCC (NRC 2015) and Eurocode 8 (CEN 2004), rely mainly on $V_{S,30}$ and $V_{S,avg}$ values, and also include the standard penetration resistance (*N*_*SPT*_) and the soil undrained shear strength (*S*_*u*_). Other classification schemes utilize the fundamental site period *T*_*0*_ (Zhao et al. 2006), or a combination of the soil thickness and stiffness properties based on $V_{S,avg}$, $V_{S,30}$, *T*_*0*_ and the thickness of the soil deposit, referred to the hybrid classification method (Pitilakis et al. 2018).

The NBCC seismic provisions, basically a replica of NEHRP (BSSC 2015), recognize five site categories ranging from A (hard rock) to E (soft soil; Table 1). They are defined mainly through correlation with *V*_*S,30*_, 30 m being the typical depth of geotechnical site investigations. Additional site class F (special soil) requiring site-specific geotechnical investigations includes liquefiable soils, sensitive or highly organic clays > 3 m in thickness, or plastic clays > 8 m thick.

**Table 1:** Standard site classifications schemes according to the NBCC and Eurocode 8

Code	Site class and *V*_*S,30*_ (m/s)				
	A	B	C	D	E
NBCC (NEHRP)	> 1500^(*)^	760–1500^(*)^	360–760	180–360	< 180
Eurocode 8	> 800^(**)^	360–800	180–360	< 180	^(***)^

*Soft soil must be < 3 m in thickness

**Surface weak materials must be < 5 m

***V_*S,avg*_ < 360 m/s and thickness 5 < *H* < 20 m

A similar approach for soil classification is suggested by Eurocode 8, since it is based on the same site parameters as NBCC. Eurocode 8 also contains five site classes from A through E though with different *V*_*S,30*_ ranges (Table 1). There is only one bedrock category, site class A, whereas site class E applies to soft soils with *V*_*S,avg*_ < 360 m/s and thickness 5 < *H* < 20 m overlying bedrock formations. Corresponding to the soil class F in NBCC, two soil types are defined, S1 and S2, for which site-specific studies have to be conducted.

On the other hand, it has been demonstrated that the fundamental site period (*T*_*0*_) can be a useful site parameter complementary to *V*_*S,30*_, as it decreases the standard deviation of residuals in the modern ground motion prediction equations (e.g., Luzi et al. 2011). The fundamental site period has been applied explicitly in site classification studies in Japan (Molas and Yamazaki 1995; Zhao et al. 2006). It can be measured in field conditions using the horizontal to the vertical spectral ratio of the ambient noise recordings (Nakamura 1989). The site classification scheme proposed by Zhao et al. (2006) contains four site classes starting from short (SC I) to long periods (SC IV). They correspond approximately to the stiffness of the soil columns defined in NBCC and NEHRP (Table 2). It should be mentioned that the hard rock sites are rock outcrops where the assessment of *T*_*0*_ is meaningless. SC I represents shallow deposit conditions with a resonance period < 0.2 s and the site-effect is similar to rock site.

**Table 2:** Site classification based on fundamental site period and corresponding NEHRP site classes (after Zhao et al. 2006)

Site class	Description	Site period	NEHRP site class
Hard rock			A
SC I	Rock	T_0_ < 0.2 s	A + B
SC II	Hard soil	0.2 ≤ *T*_0_ < 0.4 s	C
SC III	Medium soil	0.4 ≤ *T*_0_ < 0.6 s	D
SC IV	Soft soil	*T*_0_ ≥ 0.6 s	E + F

Likewise, as a part of the revision process of Eurocode 8, Pitilakis et al. (2018) proposed a novel, probably the most sophisticated classification scheme, as a combination of all main classification parameters: *T*_*0*_, *V*_*S,30*_*,* $V_{S,avg}$ and *H* (Table 3). The classificion scheme comprises six main soil classes: from A referring to rock or near rock outcrop site conditions, to X associated to special soil profiles requiring site-specific investigations. Based on the definitions, the site classes A and E are similar to the classes A and E of Eurocode 8. The difference is in the introduction of sub-classes to the general soil categories B and C, which allows for more granular analyses of the site effect in the otherwise broad representations of different soil conditions.

**Table 3:** Site classifications scheme according to Pitilakis et al. (2018)

Site class	*T*_*0*_	*V*_*S*_ (m/s)	Thickness	Description
A	≤ 0.2 s	*V*_*S30*_* or* ≥ 800	Surface weathered layer H < 5 m	Seismic bedrock
B_1_	0.1–0.3 s	$V_{s,avg}$: 350–600 *V*_*S,30*_: 400–760	H < 30 m	Very dense sand and/or stiff clay
B_2_	0.3–0.6 s	$V_{S,avg}$: 400–550 *V*_*S,30*_: 350–500	30 m < H < 120 m	
C_1_	0.6–1.0 s	$V_{S,avg}$: 400–600 *V*_*S,30*_: 350–450	H > 60 m	Medium dense sand and/or stiff clay
C_2_	0.3–0.7 s	$V_{S,avg}$: 250–450 *V*_*S30*_: 250–400	20 m < H < 60 m	
C_3_	0.7–1.4 s	$V_{S,avg}$: 300–500 *V*_*S,30*_: 200–350	H > 60 m	
D	≤ 1.4 s	$V_{S,avg}$: 200–400 *V*_*S,30*_: 150–300		Soft soil
E	0.1–0.5 s	$V_{S,avg}$: 160–300	5 m < H < 20 m	Soft soils overlaying rock (or site class A)
X	Special soils requiring site-specific evaluations ($V_{S,avg}$ < 160 m/s)			

## Materials and methods

### Geology of the study area

The presence of heterogeneous soil deposits including a soft clay layer with an important thickness and the proximity of the most active seismic zone in Eastern Canada, the Charlevoix seismic zone, prompted the selection of the city of Saguenay as a study area. Saguenay is the main urban centre within the Saguenay-Lac-Saint-Jean region and covers an area of 1136 km^2^ with a population of 147,100. It lies in the southern portion of the E-W trending Saguenay graben and is characterised by irregular topography (i.e., valleys and hills). The seismic activity of this region was reassessed following the 1988 M6.0 Saguenay earthquake. This intraplate earthquake, with a mid-crustal depth of 29 km and a moderate magnitude, occurred 35 km south of downtown Saguenay (Du Berger et al. 1991). The effects of the earthquake including soil liquefaction, rock falls, and landslides were observed as far as 200 km from the epicentre (Lamontagne 2002; Wang 2020).

**Fig. 1:**
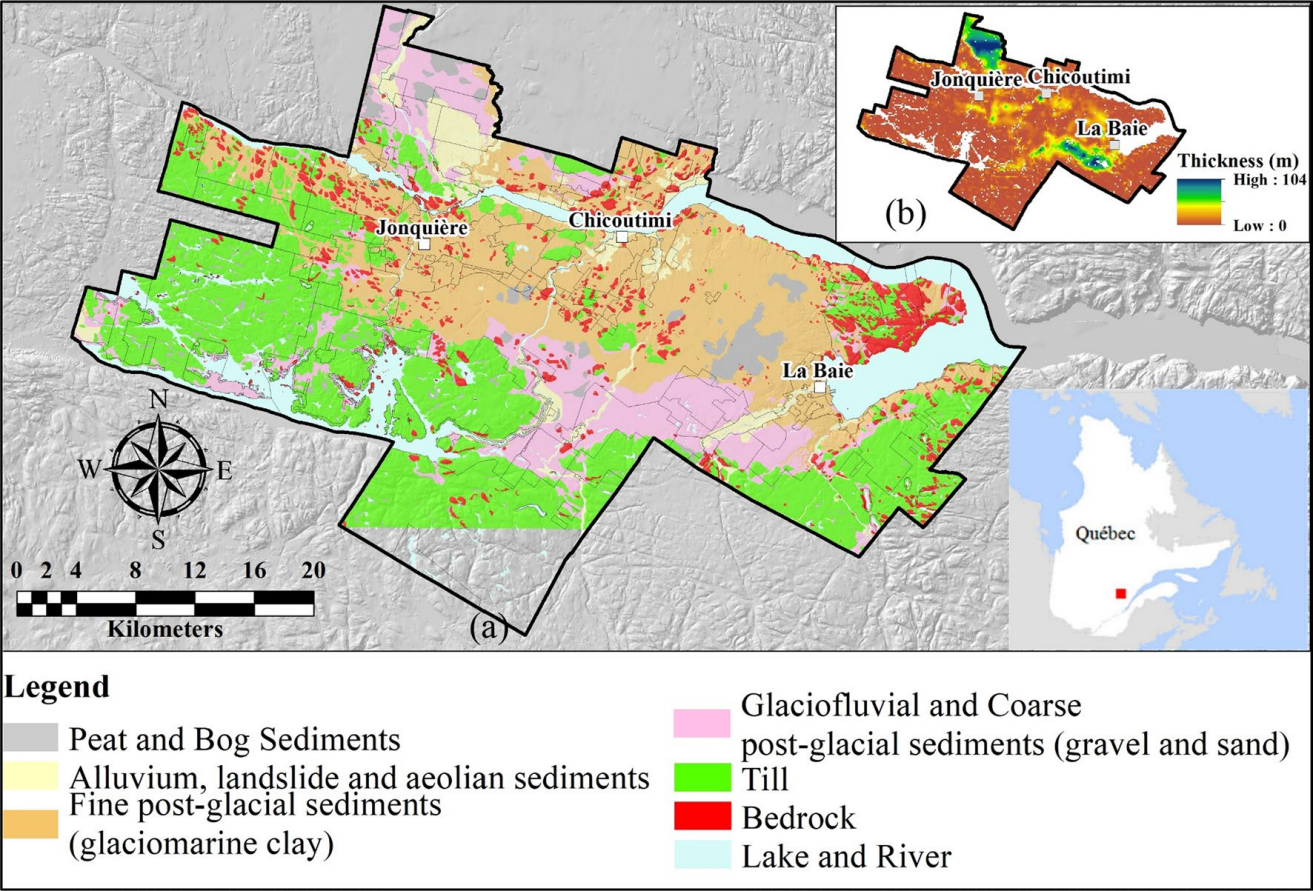
Study area; **a** simplified surface geology ( modified from Daigneault et al. 2011); **b** thickness of surficial deposits (CERM-PACES 2013)

The bedrock of this region is part of the Grenville province of the Canadian Shield and is mainly composed of crystalline Precambrian rocks (Davidson 1998). It is generally covered by recent glacial and postglacial sediments. The different stratigraphic units can be grouped into five broad categories (from bottom to surface): (1) till, (2) glaciofluvial gravel and sand, (3) fine glaciomarine sediments (clay and silt), (4) coarse glaciomarine (sand and gravel), and (5) loose postglacial deposits consisting of alluvium, floodplain sediments, organic sediments, and landslide deposits. The regional surficial geology, the total thickness of unconsolidated sediments, and the areas covered by the various units are presented in Fig. 1 and Table 4.

**Table 4:**
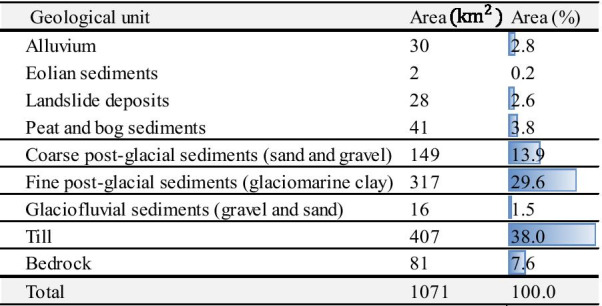
Major Quaternary units and respective coverage of the study area

The glacial till at the base of the stratigraphic column is compact, semi-consolidated, and is considered continuous in the lowlands. There, the till thickness varies from a few meters to more than 10 m in certain locations. In the highlands, the till veneer is discontinuous and alternated with frequent rock outcrops (Foulon et al. 2018). The most widespread and thickest deposits in the region are the fine postglacial sediments composed of silty clays. Bouchard and Tavenas (1983) proposed a pre-consolidation hypothesis for these clays due to the partial erosion following their deposition. These deposits are generally up to 10 m in thickness but can attain more than 100 m in certain areas in the lowlands. The remaining sedimentary units at the surface are considerably less frequent and are confined to sporadic areas in the lowlands.

### Mapping of V_S,30_, V_S,avg_ and T_0_

**Fig. 2.**
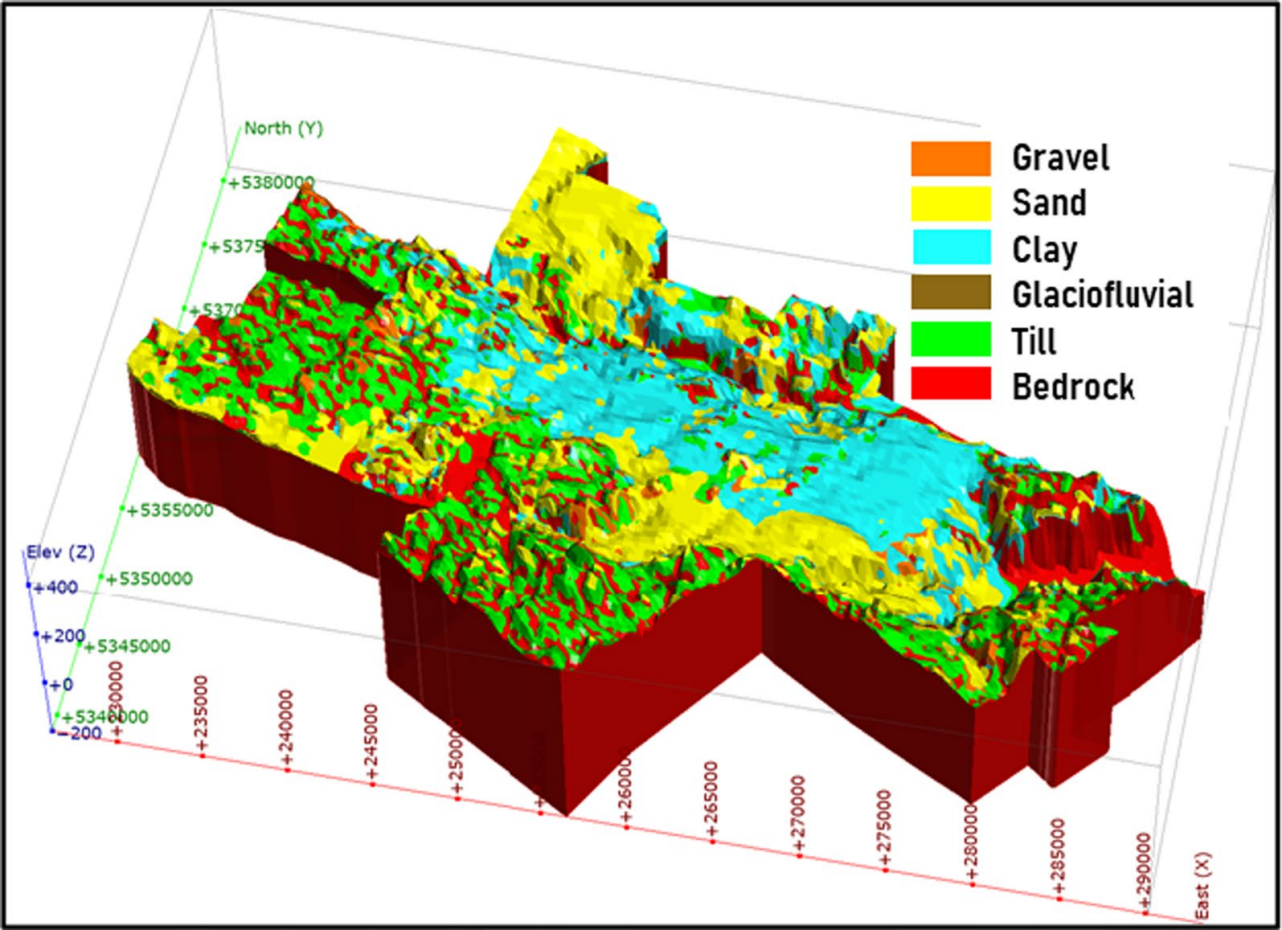
3D geological model of the study area ( modified from Foulon et al. 2018)

As discussed above, the main parameters for seismic site classification are *V*_*S,30*_*, V*_*S,avg*_ and *T*_*0*_. A 3D model of surficial geology was generated to provide valuable information on the spatial distribution of the soil units, their thickness and certain soil properties. The local geologic characteristics can be used as a proxy for estimating the shear wave velocity (Holzer et al. 2005). The accuracy of the approximation may be enhanced by obtaining the correlation between shear wave velocity and depth based on each soil type (e.g., clay, sand etc.). A 3D model provides valuable information on critical factors such as the spatial distribution of sediments, the soil properties, the thickness of the geologic units, and others. This information contributes to generating an enhanced approximation of the shear wave velocity especially for the region with sparse measured *V*_*s*_ data. Leapfrog Geo (ARANZ Geo Limited 2014) software was used herein to model the 3D stratigraphy (Fig. 2). This software uses an implicit modelling method by applying polylines (segments that define the sedimentary interfaces) and polygons representing the interfaces interpolated from the polylines. The spatial and vertical heterogeneity of the surficial sediments was modelled with five soil units based on the existing Quaternary geology maps and subsurface data interpreted from 3,342 borehole logs (Lasalle and Tremblay 1978; Daigneault et al. 2011; CERM-PACES 2013).

The adequacy of having *V*_S_ measurements from geophysical or geotechnical tests is hardly achievable for all parts of the region. By developing a *V*s-depth profile for the study area (Table 5), the shear-wave velocity variation with depth of the soil column was determined. Hence, at a location with no direct *V*S measurement, an approximate *V*_s_-value is assigned based on the *V*_s_-depth profile. Such correlations will be combined with the 3D geological model to determine the variation of *V*_s_ for each site column through the region.

**Table 5:** Representative shear-wave velocity of bedrock and surficial sediments (after Nastev et al. 2016; Foulon et al. 2018)

Location	Geological unit	Average shear-wave velocity of measurements (m/s)	Velocity-depth relationship	Remarks
Saguenay region	Sandy soils	80 ~ 260	*V*_*s*_ = 40.9 + 53.7Z^0.5^ ± 29.8 m/s	Postglacial deposits
	Clayey soils	80 ~ 250	*V*_*s*_ = 79.3 + 17.3Z^0.5^ ± 45.3 m/s	Glaciomarine clay
Ottawa and St. Lawrence Valley	Till	400 ± 152	–	Glacial deposits
	Bedrock	2500 ± 700	–	Precambrian rocks

**Fig. 3:**
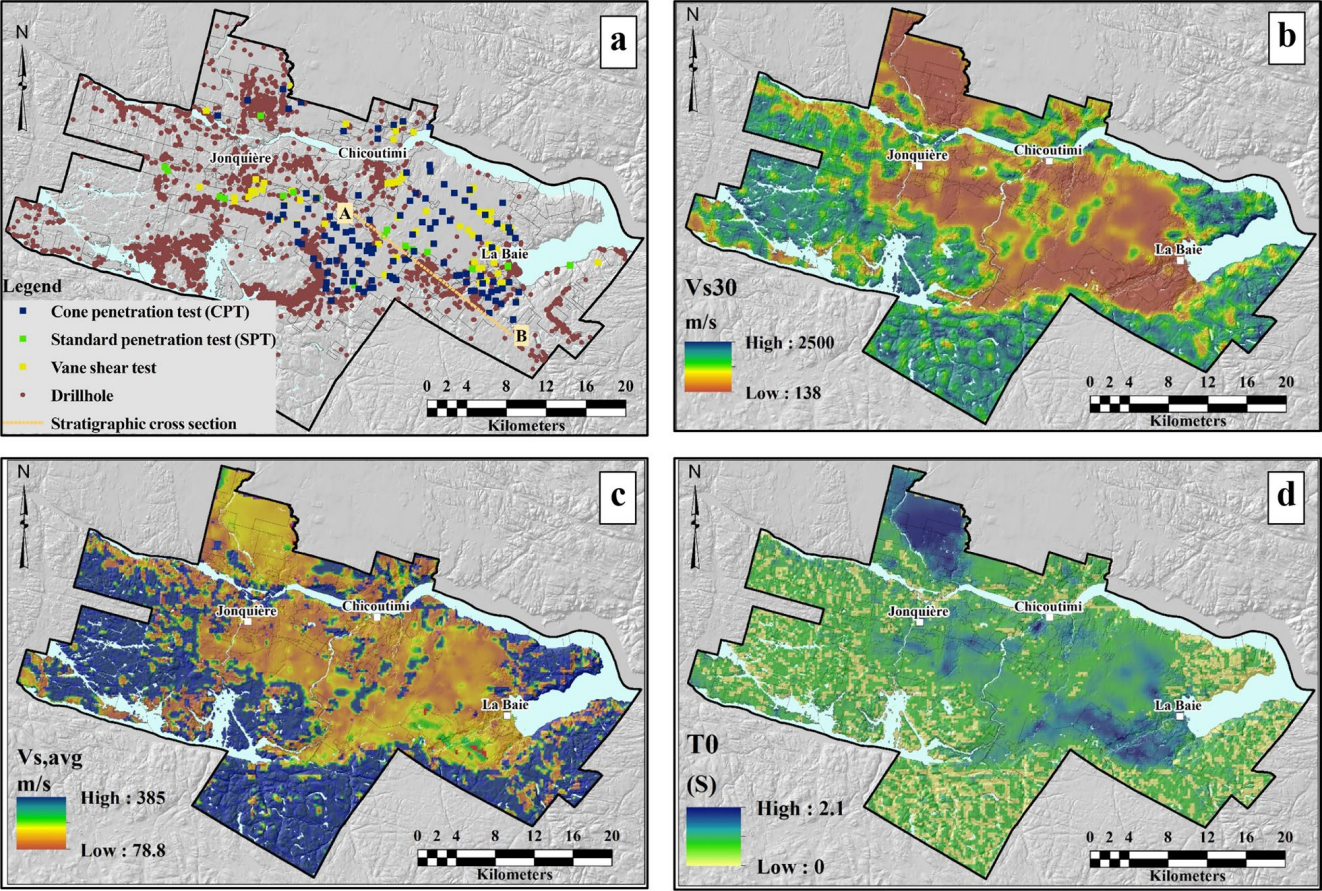
Spatial distribution of: **a** geotechnical tests and boreholes, **b** $V_{s,30}$, **c** $V_{s,avg}$ and **d** *T*_*0*_

To obtain representative interval *V*_*s*_ and *V*_*s*_-depth profiles, results of 64 standard penetration tests (SPTs) and 122 cone penetration tests (CPTs) for the Saguenay region were acquired from the Quebec Ministry of Transport (Fig. 3a). The SPT data were converted to *V*_*s*_ applying the empirical relationship of Ohta and Goto (1978) for medium sandy soils since the medium-sized sand is prevailing in the study area (Dion 1986). Meanwhile, the CPT data associated with clay deposits were converted using the empirical relationship of Mayne and Rix (1995) consistent with glaciomarine clay deposits. Due to the lack of *V*_*s*_ measurements in till deposits and bedrock, regional *V*_*s*_ estimates valid within the larger Ottawa—St. Lawrence region were applied herein (Nastev et al. 2016). The ranges of retained shear-wave velocities and standard deviations for selected Quaternary deposits are given in Table 5.

The mapping of *V*_*S,30*_, *V*_*S,avg*_ and *T*_*0*_ was performed using the 3D geological model and the representative *Vs*-depth functions (Fig. 3b, c and d). Appropriate *V*_*s*_ was $V_{S,avg}$ assigned to each unit based on the depth and the soil type. Then, the averaged values were calculated on a 2D raster with a cell size of 250 × 250 m. The *V*_*S,30*_ and *V*_*S,avg*_ values for each cell were calculated using the following equations,

1 $$V_{S,30} = \frac{30}{{\left( {\mathop \sum \nolimits_{i = 1}^{n} \left( {\frac{{h_{i} }}{{V_{{s_{i} }} }}} \right) + \frac{{(30 - \mathop \sum \nolimits_{i = 1}^{n} h_{i} )}}{{V_{{s_{rock} }} }}} \right)}}$$

2 $$V_{S,avg} = \frac{{\text{H}}}{{\left( {\mathop \sum \nolimits_{i = 1}^{n} \left( {\frac{{h_{i} }}{{V_{{s_{i} }} }}} \right)} \right)}}$$

where $h_{i}$ and $V_{{s_{i} }}$ are the thickness and the interval shear-wave velocity of each layer *i*, respectively. The bedrock shear-wave velocity, *V*_*s,rock*_, was included for deposit thickness lower than 30 m. The exception was cells where the soft soil thickness was more than 3 m and the *V*_*S,30*_ calculated initially was higher than 760 m/s. In this case *V*_*S,30*_ was substituted with *V*_*S,avg*_ of soils. Alternatively, *T*_*0*_ and subsequent harmonics were approximately estimated with the theoretical solution for a vertically propagating horizontal shear-wave in elastic homogeneous soils given with the following equation (Kramer 1996):

3 $$T_{{\text{n}}} = \frac{4 \times H}{{V_{S,avg} \times \left( {1 + 2n} \right)}},\;for\;n = 0,1,2 \ldots ,$$

where H is the total soil thickness and n ≥ 1 indicates higher harmonics.

## Results and discussion

### Site classification results

**Fig. 4:**
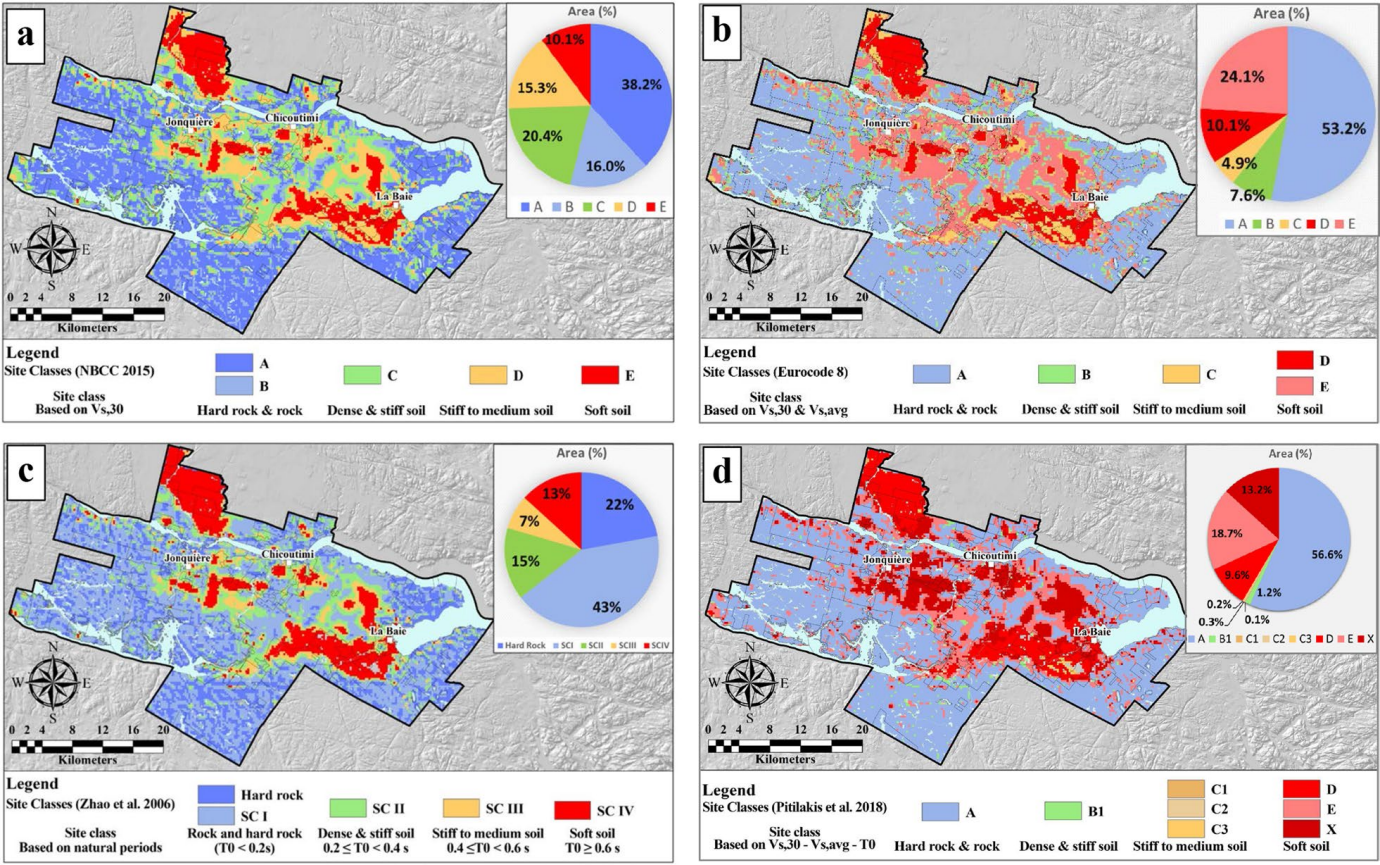
Spatial distribution of seismic site classes and relative coverage of the study area based on site classification methods: **a** NBCC, **b** Eurocode 8, **c** fundamental periods of soil (*T*_*0*_) and **d** hybrid approach

The regional seismic site classification was conducted based on the NBCC, Eurocode 8, the fundamental site period (Zhao et al. 2006), and the hybrid approach (Pitilakis et al. 2018). The results of the site classifications are given in Fig. 4. General observations are discussed below for each of the applied site classification methods.

*NBCC* (Fig. 4a): rock site conditions A and B cover 38.2% and 16.0% of the study area, respectively, which is actually more than 50% of the mapped area. These site classes are found in rock outcrops and areas where bedrock is covered with till veneer or with soft sediments less than 3 m thick. There, the ground surface practically replicates the bedrock surface. Site classes C and D share 20.4% and 15.3% of the study area, respectively, whereas only 10.1% of the study area is classified as soft soil, site class E. Due to the predominantly shallow sediments conditions in the region and the addition of the high *V*_*Srock*_ in the *V*_*S,30*_ assessment, site classification based on NBCC ends up in about 90% of the area being classified as rock and dense stiff soil. To evaluate the effect of averaging the *V*_*S,30*_ across geologic units with significantly different *V*_*s*_, the contribution of the individual units in each site class is calculated from the 3D geological model. The influence of the contributing thicknesses in terms of percentage can then be considered as representative of the regional impact of a given geologic unit to each site class (Table 6).

**Table 6:** Contribution of the different geologic units to the NBCC site classes

Site class	% of total area	Bedrock (%)	Till (%)	Glaciofluvial sediments (%)	Clay (%)	Sand and gravel (%)	Total (%)
A	38.2	97.5	2.5	0.0	0.0	0.0	100.0
B	16.0	86.4	11.0	0.0	1.4	1.1	100.0
C	20.4	76.3	7.4	0.4	8.4	7.5	100.0
D	15.3	39.7	8.6	3.6	28.2	20.0	100.0
E	10.1	2.4	3.5	2.2	69.9	21.9	100.0

It can be observed in Table 6 that bedrock is the dominant unit in site classes A to D in absolute or relative terms, with contributions varying from 39.7% for site class D to 97.5% for site class A. Of particular concern is the significant contribution of *V*_*Srock*_ in site classes C and D. This occurs in areas with relatively shallow unconsolidated sediments, where clayey soils are the second most important unit. There, the high impedance contrast may lead to seismic amplification considerably higher than otherwise predicted by NBCC. The thick clay sediments are by far the major contributor to the site class E with practically negligible participation of the bedrock, 2.4%.

*Eurocode 8* (Fig. 4b): in this site classification, 53.2% of the area is delineated as rock, site class A. Similar to the NBCC classification, rock sites cover more than half of the study area comprising bedrock or shallow till outcrops. The spatial coverage of site classes C and B, stiff to very stiff soils, corresponding to NBCC site classes C and D, decreases considerably to 7.6% and 4.9%, respectively. Soft soil, ground type D, covers 10.1% of the study area, whereas 24.1% was delineated as ground type E, which represents less than 20 m thick soil column overlying bedrock. Again, the influence of the contributing thickness of each geologic unit was considered in the estimation of *V*_*s30*_ and *V*_*s,avg*_ (Table 7). The contribution of the bedrock thickness is limited practically to site classes A and B only, whereas sands and gravels dominate in the site class C and clayey sediments in the site class D. Based on the definition of soil type E, the bedrock impacts are excluded in areas with shallow surficial sediments.

**Table 7:** Contribution of the different geologic units to the Eurocode 8 site classes

Site class	% of total area	Bedrock (%)	Till (%)	Glaciofluvial sediments (%)	Clay (%)	Sand and gravel (%)	Total (%)
A	53.2	94.5	4.8	0.0	0.4	0.3	100.0
B	7.6	81.6	7.4	0.1	6.2	4.7	100.0
C	4.9	14.4	11.6	8.4	22.6	43.1	100.0
D	10.1	2.4	3.5	2.2	69.9	21.9	100.0
E*	24.1	–	21.5	2.4	51.7	24.4	100.0

* *V*_*S,avg*_ estimated

*Fundamental site period* (Fig. 4c): the main portion of the region is identified as rock outcrop (22%) and site class *SCI* (43%) with an average vibration period less than 0.1 s (Table 8). The site response there will coincide with the seismic energy content at high frequencies. Site class *SCII* covers 15% of the study area and corresponds to relatively softer, *V*_*S,avg*_ = 142 m/s, and shallower soils, H = 10.3 m. The site classes *SCIII* and *SCIV*, on the other hand, are with similar *V*_*s,avg*_, but with significantly higher average thickness confirming that the thickness is more important for the determination of *T*_*0*_ than *V*_*S,avg*_ (the denominator in Eq. 1). It can also be observed in Table 8 that *V*_*S,30*_ is inversely proportional and correlates better to *T*_*0*_ values than the average *V*_*S*_ values*.*

**Table 8:** Descriptive statistical parameters of site classes based on fundamental periods

Site class	% of total area	Mean *T*_*0*_ (s)	Mean *V*_*S,avg*_ (m/s)	Mean *V*_*S,30*_ (m/s)	Mean thickness (m)
Bedrock	22	–	–	2500	–
SCI	43	0.07	274	1304	3.6
SCII	15	0.29	142	400	10.3
SCIII	7	0.49	151	245	18.6
SCIV	13	1.03	176	165	46.3

*Hybrid approach* (Fig. 4d): as expected, most of the study area is delineated as rock or near rock outcrop conditions, site class A. Note that the influence of the bedrock is excluded herein due to the application of *V*_*s,avg*_ and *T*_*0*_ in the site classification. The spatial coverage of stiff and dense soils, site classes B_1_ and C_1_, C_2_ and C_3_ combined is almost negligible with only about 2% (Table 9). For comparison, the corresponding NBCC classes C and D have significantly higher coverage, 35.8%, as a consequence of adding *V*_*Srock*_ in the *V*_*S,30*_ estimates. Softer soils are considerably more represented: site classes D and E cover 9.6% and 18.7% of the study area, respectively, whereas about 13.2% are classified as special soils which require site-specific evaluation, site class X. Till is the main geological unit in the determination of site classes A and B, sands and gravels are dominant in the site class C, whereas site classes D, E and X consist predominantly of clayey soils.

**Table 9:** Total thickness percentage of geological units contributing to the estimation of site classes based on the hybrid classification approach

Site Class	% of total area	Bedrock	Till (%)	Glaciofluvial sediments (%)	Clay (%)	Sand and gravel (%)	Total (%)
A	56.6	–	73.31	0.14	13.82	12.73	100.00
B1	1.2	–	99.96	0.00	0.00	0.04	100.00
C1	0.1	–	20.16	11.87	0.20	67.78	100.00
C2	0.3	–	33.99	2.32	3.22	60.47	100.00
C3	0.2	–	14.63	4.61	21.45	59.31	100.00
D	9.6	–	12.37	7.37	55.76	24.51	100.00
E	18.7	–	28.03	3.07	31.92	36.98	100.00
X	13.2	–	10.65	0.94	79.37	9.05	100.00

### Comparative analysis

The correlations between the site parameters are analysed in this chapter to better understand the causes for eventual discrepancies.

#### Statistical comparison

**Fig. 5:**
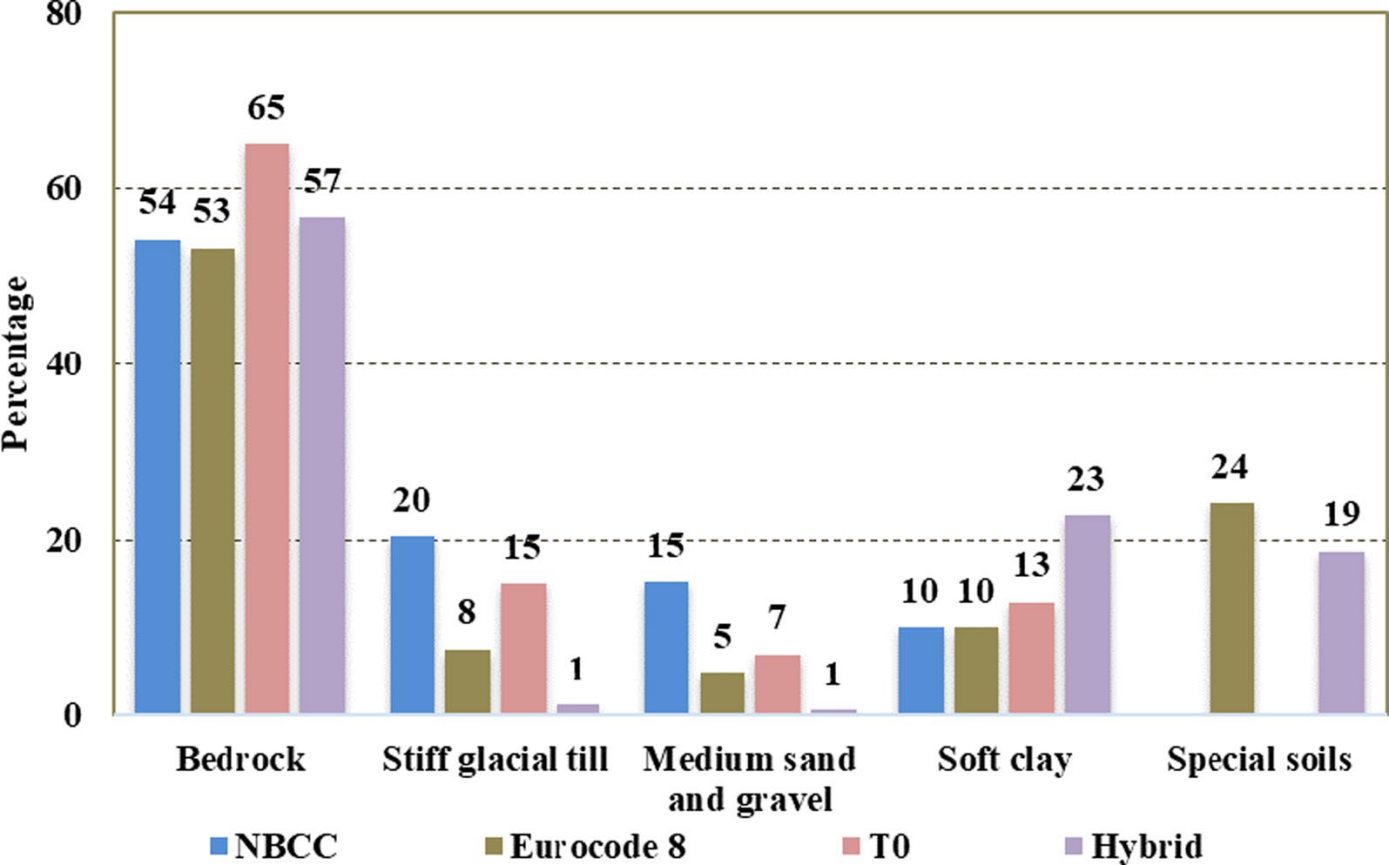
Relative surface area coverage by each soil type based on the four site classification schemes: Eurocode 8, NBCC, the fundamental site period and the hybrid method

The simplified geology of the study area comprises Precambrian bedrock, stiff dense glacial till, and post-glacial sandy, soft clayey soils and other soft soils which require site-specific study. Each of these four main geological units and the special soils has its own range of $V_{s,30} ,V_{s,avg}$ and *T*_*0*_, which result in different site categories depending on the applied site classification method. In order to assess the efficiency of the seismic microzonation methods to recognize these geologic conditions, the resulting site classes are compared statistically and differences are quantified in percentages (Fig. 5).

Figure 4 shows that all four microzonation methods appear to be in relatively good agreement for bedrock and sites with dominant soft clayey soils. For mainly stiff and soils with medium stiffness, the NBCC and *T*_*0*_ methods are well correlated with each other. On the other hand, the Eurocode 8 and the hybrid classifications underestimate the stiff and medium soils conditions sorting them either as soft or special soils (site class E). The areas covered with these soil types produce the main site classification difference between the NBCC and *T*_*0*_ methods on one side and Eurocode and the hybrid method on the other.

#### Geological cross-section

**Fig. 6:**
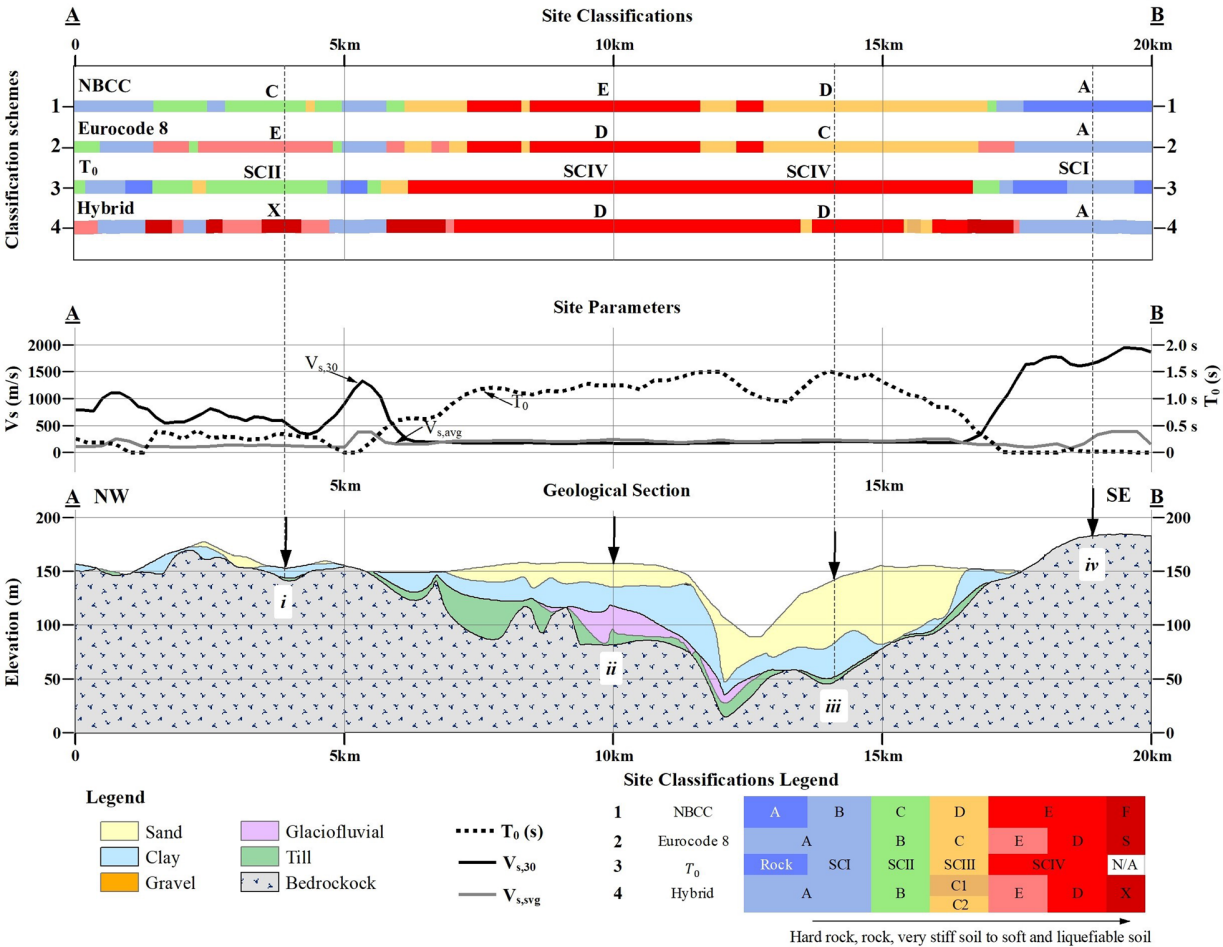
Representative cross-section (from below): local geology with the four stratigraphic columns (i through iv), variation of the three main seismic parameters (*V*_*s,30*_,* T*_*0*_, *V*_*s,avg*_), and the seismic site classification based on the four different schemes. The location of the cross-section is indicated in Fig. 3a

Another comparison was conducted over a representative 20 km long cross-section taken as an example of the geological setting in the study area together with four typical stratigraphic columns indicated with i through iv in Fig. 6. As can be observed, the thickness and soil types vary laterally and vertically suggesting different site class evaluations. Thanks to the geological and geotechnical profiles, the similarities and differences of each site classification scheme are better compared:

(i) Medium thickness sediments, 5 < H < 30 m: The site evaluations are different in this stratigraphic column. NBCC and *T*_*0*_ site classification methods identify rock and stiff soil conditions. On the other hand, for Eurocode 8 and the hybrid method these site conditions are soft soils since they both take into account *V*_*S,avg*_ of the surficial sediments instead of *V*_*S,30*_ and *T*_*0*_ by the former two methods.
(ii) Thick sediments, H > 30 m: the observed evaluations of site conditions are in fair agreement, soft soils, by all four classification methods.
(iii) Thick sediments, H > 30 m: the *V*_*S,30*_ based site classifications, NBCC and Eurocode 8, evaluate this soil column with medium stiffness, whereas *T*_*0*_ and the hybrid method identify rather soft soil conditions. This is because, in the two former schemes, the soil thickness has more weight in the final results than the stiffness (*V*_*s*_). A typical example is the differences in site classification of stratigraphic columns ii and iii due to the presence of thick stiff sandy soils.
(iv) Shallow sediments, H < 5 m: this geological setting is probably the least challenging and all site classification methods are in agreement evaluating rock or shallow rock site conditions with a short fundamental period (*T*_*0*_ < 0.2 s).

#### Correlations of seismic site parameters

**Fig. 7:**
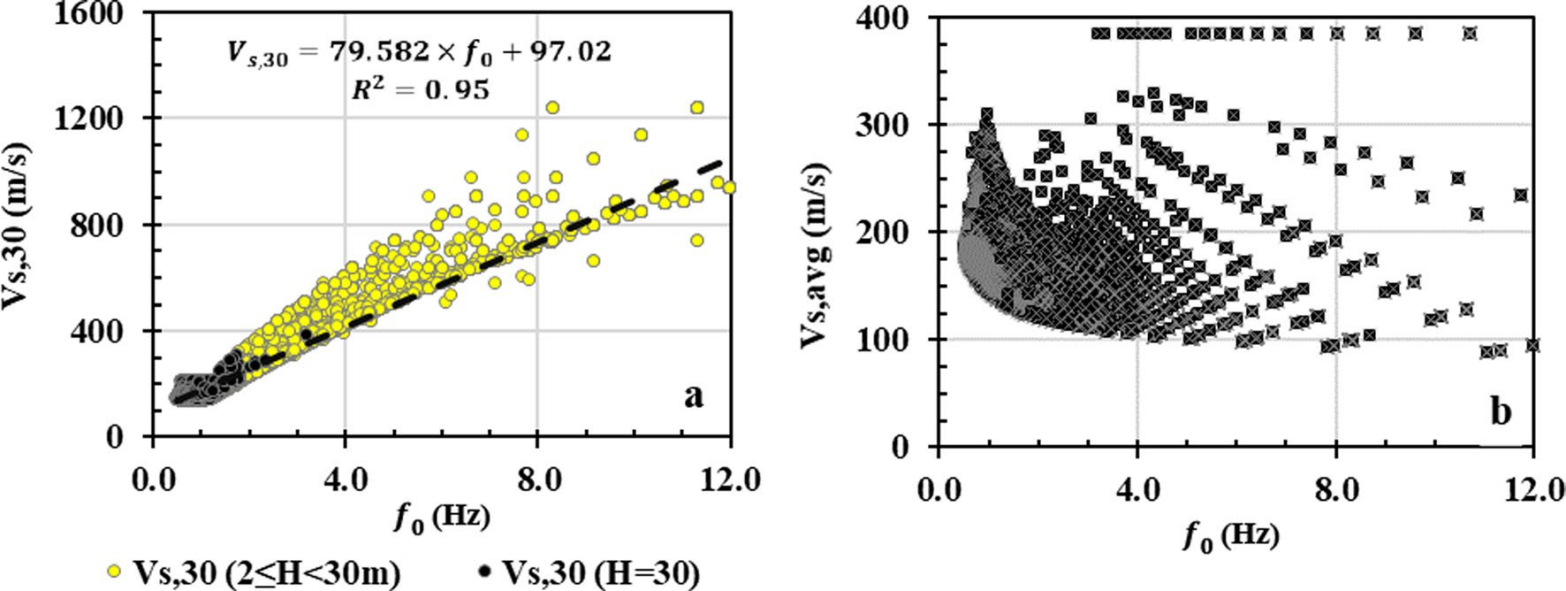
Correlation of the fundamental site frequency *f*_*0*_ with **a** *V*_*S,30*_, and **b** *V*_*S,avg*_

Of particular interest are the mutual relationships between the main site parameters. Understanding the correlations between the site parameters helps identify the similarity and differences in the respective site classification results. It can also help to eliminate well-correlated parameters and retain those that represent best the local site conditions, thus reducing the complexity of the site classification without omitting valuable information. In this respect, regression analyses are conducted between *V*_*S,30*_, *V*_*S,avg*_ and the fundamental site frequency, *f*_*0*_ = 1/*T*_*0*_ (Fig. 7). The results reveal a strong correlation, R^2^ = 0.95, between *V*_*S,30*_ and *f*_*0*_ for 9,246 measurement sites (Fig. 7a). Simple linear regression is applied for frequencies of up to 12 Hz, since data are sparse beyond this limit. To better understand the influence of the *V*_*s,rock*_ on the correlation, data for surficial soil with *H* < 30 m are represented with yellow dots. At these sites, the *V*_*S,30*_ is estimated as a combination of *V*_*s,rock*_ and *V*_*s*_ of soils. Elsewhere, *V*_*S,30*_ corresponds to deeper soil sediments with H > 30 m, indicated with black dots (left hand side of Fig. 7a). On the other hand, it can be observed in Fig. 7b that the correlation between *V*_*S,avg*_ and *f*_*0*_ is practically inexistent. This suggests that the addition of *V*_*S,rock*_ to the soil *V*_*s*_ in the top 30 m actually improves the correlation with *f*_*0*_. A similar conclusion on the relationship between *V*_*S,30*_ and *f*_*0*_ was obtained in site amplification studies by Ghofrani et al. (2013) and Finn and Ruz (2015). These studies showed a strong *V*_*S,30*_ vs. *f*_*0*_ correlation in shallow soils, which was not necessarily observed in deep soil sediments characterised with low frequencies.

## Conclusion

A seismic microzonation study was conducted in the complex geologic environment underlying the city of Saguenay. The main seismic site classification parameters considered in the analyses were: average shear-wave velocity of the top 30 m (*V*_*s,30*_), average shear-wave velocity for the total thickness of the surficial sediments (*V*_*s,avg*_) and the fundamental site period (*T*_*0*_). Four different classification schemes were applied and compared: NBCC, Eurocode 8, fundamental site period and the hybrid approach based on all main site parameters. All of the classification methods have their own advantages and limitations with respect to the local geological and geotechnical conditions. The following are the major conclusions drawn from this study:

- The site classifications based on *V*_*S,30*,_ NBCC and Eurocode 8, appear the most consistent with the local conditions. The results could be further improved considering secondary parameters, e.g., *V*_*S,avg*_ in shallow (H < 30 m) and *T*_*0*_ in deeper soil sediments (H > 30 m). In such a case, the impact of the stiffness and thickness of the surficial sediments will be better accounted for.
- Site class E in Eurocode 8 and the hybrid method refers to medium to soft soils, 5 < H < 20 m, on top of bedrock. In NBCC, the *V*_*S*_ of these soils is combined with *V*_*Srock*_ yielding V_S,30_ considerably higher than *V*_*S*_. Since such site conditions cover a significant portion of the study area, their dynamic response should be analysed more in detail.
- Eurocode 8 doesn’t include the hard-rock site condition as is the case with the site class A in NBCC. Classification of rock sites into two categories helps distinguish the site effect in crystalline hard rocks and more fractured sedimentary rocks formations.
- A strong correlation between *V*_*S,30*_ and *T*_*0*_ was observed in shallow sediments (H < 30 m) and a relatively weaker correlation in deeper sediments (H > 30 m), whereas the correlation between *V*_*s,avg*_ and *T*_*0*_ is practically inexistent. This suggests that the addition of *V*_*s,rock*_ to the *V*_*s*_ soil in the top 30 m improves the correlation between *V*_*s,30*_ and *T*_*0*_. Due to this strong correlation, NBCC site classification yields similar patterns as the *T*_*0*_ scheme.
- The site classification based on *T*_*0*_ is affected considerably more by the thickness of the overlying sediments than by *V*_*S,avg*_. Therefore, most of the shallow deposit conditions have a resonance period < 0.4 s, which highlights the potential for seismic amplification in the short period range.
- The hybrid site classification proposes a multitude of classification parameters, which, in certain cases, may lead to confusion in selecting the appropriate site class. However, the results arrange the site conditions mainly into two major groups: rock and soft soils. Stiff and medium stiffness soils share only a limited part of the study area as opposed to the NBCC site classification.

As an overall conclusion, this study demonstrates that site classification based on *V*_*s,30*_ is in general consistent with the geological and geotechnical conditions of the study area. However, the results may be further improved considering *V*_*s,avg*_ in shallow (H < 30 m) and *T*_*0*_ in deeper soil sediments (H > 30 m) as secondary parameters. In such a case, the impact of the stiffness and thickness of the surficial sediments will be better accounted for.

## Data Availability

All the datasets that have been used and analysed during the current study is available from the corresponding author on reasonable request.

## List of symbols

CPT: Cone penetration test
*f*_*0*_: Fundamental site frequency
*H*: Soil thickness
NBCC: National Building Code of Canada
SPT: Standard penetration test
*T*_*n*_: Natural period of vibration
*T*_*o*_: Fundamental site period of vibration
*V*_*s*_: Shear wave velocity
*V*_*s*__,30_: Average shear-wave velocity of the top 30 m
*V*_*S*__,__*avg*_: Average shear-wave velocity of the entire soil deposit
*V*_*s,rock*_: The bedrock shear-wave velocity
